# Structure and Spectroscopic Insights for CH_3_PCO Isomers: A High-Level Quantum Chemical Study

**DOI:** 10.1021/acs.jpca.4c01370

**Published:** 2024-05-09

**Authors:** Miguel Sanz-Novo, Pilar Redondo, Clara Isabel Sánchez, Antonio Largo, Carmen Barrientos, José Ángel Sordo

**Affiliations:** † Centro de Astrobiología (CAB), INTA-CSIC, Carretera de Ajalvir km 4, Torrejón de Ardoz, 28850 Madrid, Spain; ‡ Departamento de Química Física, Universidad de Valladolid, 47011 Valladolid, Spain; § Departamento de Química Física y Analítica, Laboratorio de Química Computacional, Facultad de Química, Universidad de Oviedo, Julián Clavería 8, 33006 Oviedo, Principado de Asturias, Spain

## Abstract

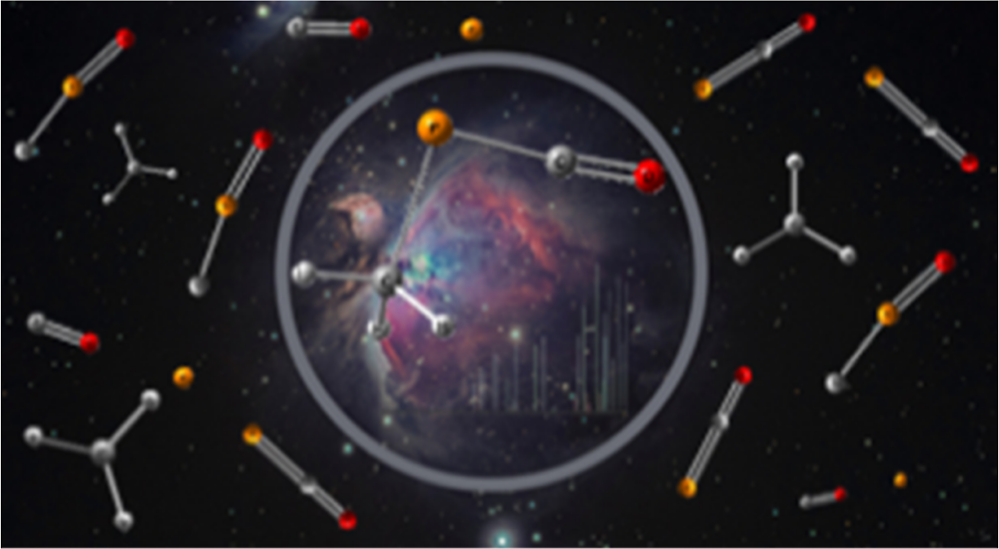

The exploration of phosphorus-bearing species stands as a prolific field in current astrochemical research, particularly within the context of prebiotic chemistry. Herein, we have employed high-level quantum chemistry methodologies to predict the structure and spectroscopic properties of isomers composed of a methyl group and three P, C, and O atoms. We have computed relative and dissociation energies, as well as rotational, rovibrational, and torsional parameters using the B2PLYPD3 functional and the explicitly correlated coupled cluster CCSD(T)-F12b method. Based upon our study, all the isomers exhibit a bent heavy atom skeleton with CH_3_PCO being the most stable structure, regardless of the level theory employed. Following in energy, we found four high-energy isomers, namely, CH_3_OCP, CH_3_CPO, CH_3_COP, and CH_3_OPC. The computed adiabatic dissociation energies support the stability of all [CH_3_, P, C, O] isomers against fragmentation into CH_3_ and [P, C, O]. Torsional barrier heights associated with the methyl internal rotation for each structure have been computed to evaluate the occurrence of possible *A*–*E* splittings in the rotational spectra. For the most stable isomer, CH_3_PCO, we found a *V*_3_ barrier of 82 cm^–1^, which is slightly larger than that obtained experimentally for the N-counterpart, CH_3_NCO, yet still very low. Therefore, the analysis of its rotational spectrum can be anticipated as a challenging task owing to the effect of the CH_3_ internal rotation. The complete set of spectroscopic constants and transition frequencies reported here for the most stable isomer, CH_3_PCO, is intended to facilitate eventual laboratory searches.

## 1 Introduction

Phosphorus (P) is a second-row element that is vital for the development of life. Yet, it exhibits a relatively low cosmic abundance compared to other biogenic elements such as hydrogen (H), carbon (C), oxygen (O), nitrogen (N), and sulfur (S) (*e.g.*, the solar P/H ratio is ∼3 × 10^–7^).^1^ Phosphorus plays a crucial role in living organisms,^2^ where its abundance is much higher (*e.g.*, P/H ∼ 10^–3^ in bacteria).^3^ It is a fundamental component of relevant biomolecules such as phospholipids (cellular membrane components), and adenosine triphosphate (ATP), being responsible for storing chemical energy in cells. Moreover, it is thought to have been initially brought to Earth by the impact of meteorites.^2,4^

Despite its low cosmic availability, to date, seven P-containing molecules (PN, CP, HCP, PO, PO^+^, C_2_P, and PH_3_) have been conclusively found in the interstellar medium (ISM) or circumstellar shells,^5−12^ highlighting the recent detection of phosphorus monoxide (PO) and phosphorus nitride (PN) at the edge of the Galaxy.^13^ Phosphorus was also detected throughout the Rosetta mission in the comet 67P/Churyumov–Gerasimenko,^14^ which is mainly in the form of PO.^15^ More recently, in 2022, the tentative detection of the SiP radical in the circumstellar shell of IRC +10216 has been reported.^16^ It was argued that SiP could be obtained from grain destruction releasing P and Si into the gas phase and then it is addressed to other P-bearing molecules. In this context, the limited number of detected P-bearing species motivates the astrochemical community to propose new P-bearing interstellar candidates to expand the knowledge on the chemistry of phosphorus in the ISM.^17−21^

On a different note, isocyanates [*i.e.*, molecules containing the isocyanate (−N=C=O) functional group] are of prebiotic relevance due to their role in amino acid synthesis, peptide polymerization,^22^ and nucleotide/nucleoside production.^23^ Until now, only five isocyanates have been detected in the ISM: (i) isocyanic acid (HNCO);^24−34^ (ii) its cationic form, H_2_NCO^+^;^35,36^ (iii) the NCO radical,^36^ which stands as the simplest molecule containing the backbone of the peptide bond; (iv) methyl isocyanate (CH_3_NCO);^34,37−44^ and (v) ethyl isocyanate (CH_3_CH_2_NCO).^45^

Methyl isocyanate CH_3_NCO was first detected in 2015 both in volatized material from comet 67P/Churyumov–Gerasimenko by Rosettaʼs Philae lander^46^ and in the 3 mm segment of a broadband survey of Sgr B2(N).^37^ Since then, CH_3_NCO has been identified in several interstellar sources such as Orion clouds,^38^ toward the low-mass protostar IRAS 16293-2422^39^ or in a solar-type protostar, IRAS 16293-2422 B.^40^ Consequently, the isovalent phosphorus analogs of the above systems emerge as appealing candidates for interstellar detection, even though most of them remain uncharted in the laboratory. Therefore, in this work, we report a state-of-the-art theoretical study for the most stable [CH_3_, P, C, O] isomers: CH_3_PCO (global minimum in energy), CH_3_OCP, CH_3_CPO, CH_3_COP, and CH_3_OPC. We first explore their structure and stability, including dissociation processes for all the above structures, and investigate the barrier to a plausible methyl internal rotation. Moreover, we provide a high-level calculated set of rotational spectroscopic constants for the most stable CH_3_PCO isomer, the phosphorus analog of the interstellar species methyl isocyanate, which will be of great need to conduct spectral searches in the laboratory and serve as relevant benchmark data to compare against experimental information once available. This information shall help us to add another piece to the enigma of the astrochemistry of phosphorus.

## 2 Computational Methods

We have employed *ab initio* and density functional theory (DFT) methodologies in the study of the [CH_3_, P, C, O] isomers. First, we characterized the different isomers on the singlet potential energy surface (PES) using DFT, in particular, the double-hybrid B2PLYPD3 functional.^47^ This functional includes Hartree–Fock exchange and a perturbative second-order correlation part as well as Grimme’s D3BJ empirical dispersion term.^48^ The Dunning’s correlation consistent triple-ζ basis set, aug-cc-pVTZ, which includes both polarization and diffuse functions on all elements, was employed in conjunction.^49,50^ Afterward, explicitly correlated coupled cluster theory with single and double excitations including triplet excitations through a perturbative treatment, CCSD(T)-F12b,^51^ in conjunction with the cc-pVTZ-F12 basis set,^52^ was employed to compute more precise structural parameters and energies on the previously located stationary points. In a recent study,^53^ we have shown that CCSD(T)-F12b/cc-pVTZ-F12-optimized structures and energies are in excellent agreement with that obtained employing a “composite” scheme (where, starting from the CCSD(T)/cc-pVTZ results, corrections are added for basis set truncation error, diffuse function, and core–valence correlation) with a lower computational cost.

Harmonic vibrational frequencies were calculated at the B2PLYPD3 and CCSD(T)-F12b level to characterize the optimized structures as minima (all real frequencies) and to obtain their zero point vibrational (ZPV) energy.

For the most stable isomer harmonic vibrational frequencies were calculated at the CCSD(T)/aug-cc-pVTZ level. Anharmonic corrections were estimated at the CCSD(T)/cc-pVTZ level of theory using the second-order vibrational perturbation theory (VPT2)^54^ within the context of the Watson Hamiltonian.^55^ A full cubic force field (CFF) and semidiagonal quartic force constants have been included in the procedure. From the CFF calculations, we have computed vibration–rotation interaction constants. The results help in the identification of the most stable [CH_3_, P, C, O] isomer by infrared spectroscopy.

Additionally, we have characterized the nature of the bonding of [CH_3_, P, C, O] isomers through a topological analysis of the electronic density in the framework of Bader’s quantum theory of atoms in molecules QTAIM^56^ using the AIMAll package^57^ including standard thresholds.

All electronic structure computations, required as inputs for the rotational and vibrational analysis, were performed in the framework of quantum mechanics methodologies as implemented in the Gaussian 16 program package,^58^ MOLPRO,^59^ and the CFOUR program.^60^

## 3 Results and Discussion

### 3.1 Structure and Stability of the Most Relevant [CH_3_, P, C, O] Isomers

**Figure 1:**
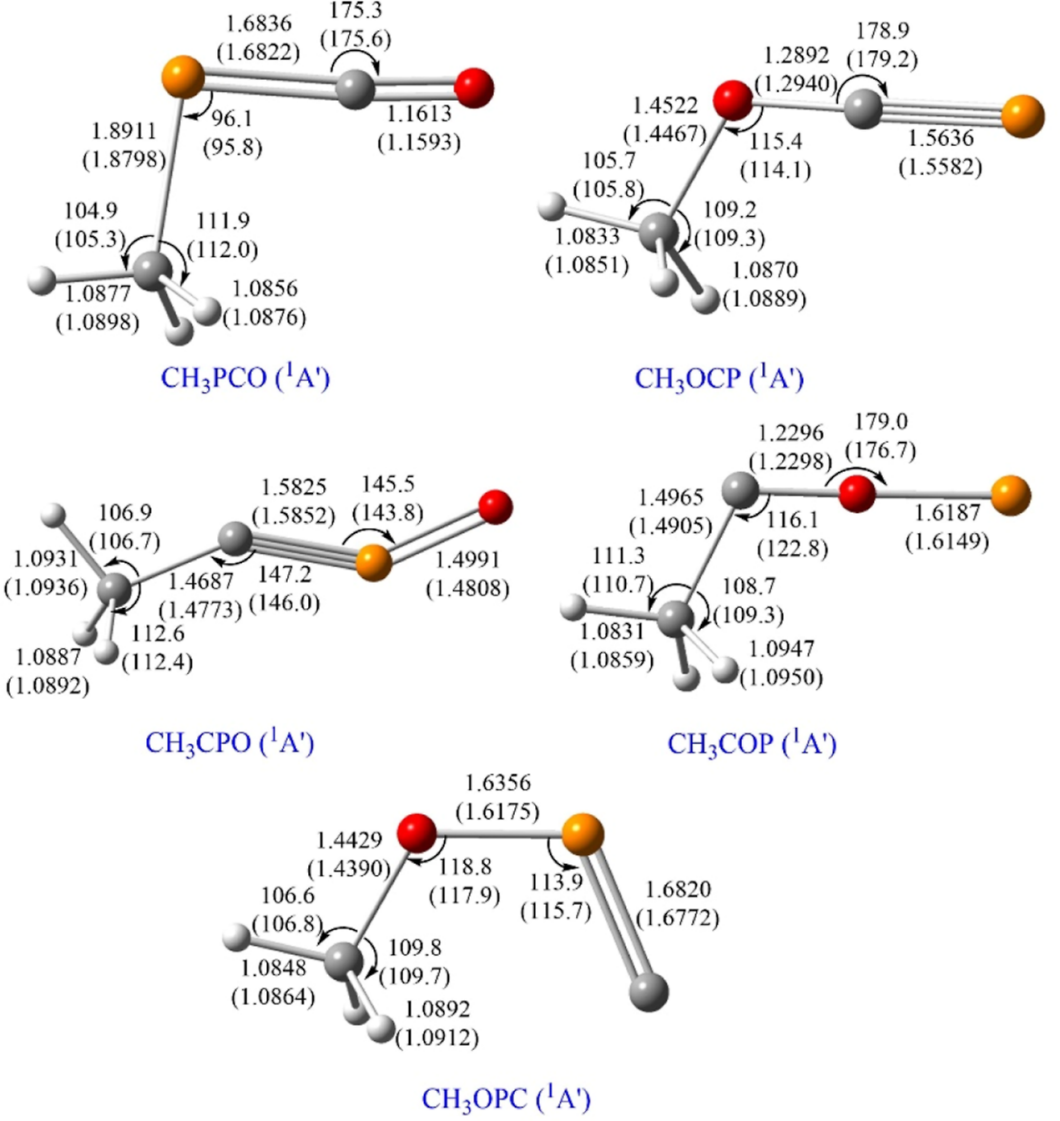
Optimized geometrical parameters (in angstroms and degrees) of the [CH_3_, P, C, O] isomers located at the B2PLYPD3/aug-cc-pVTZ and CCSD(T)-F12b/cc-pVTZ-F12 (in parentheses) levels of theory. Color code: carbon atoms are depicted in gray; oxygen atoms are in red; phosphorus atoms are in orange and hydrogen atoms are in white.

We performed a preliminary search for all plausible structures obeying the formula [CH_3_, P, C, O] at the B2PLYPD3/aug-cc-pVTZ level of theory and located five distinct structures as true energy minima: CH_3_PCO, CH_3_OCP, CH_3_CPO, CH_3_COP, and CH_3_OPC. *A priori*, we expected an additional structure, CH_3_POC, which was not found to be stable on the singlet potential energy surface. We have explored the conformational panorama of each isomer. When different conformers are possible on the corresponding PES, we have included exclusively the most stable one. However, we only found an additional *trans*-conformer for CH_3_OPC, which is located 5.8 kcal mol^–1^ above the *cis*-form at the B2PLYPD3/aug-cc-pVTZ level. We then refined the geometry and energetic calculations using the CCSD(T)-F12b method in conjunction with the cc-pVTZ-F12 basis set. We present in Figure 1 the structural parameters of the five CH_3_PCO isomers, computed at the aforementioned levels of theory. Also, Table 1 collects their relative energies (including ZPV energies), equilibrium rotational constants, and dipole moment components. Both B2PLYPD3 and CCSD(T)-F12b results yield similar outcomes for the most stable CH_3_PCO and CH_3_OCP isomers, whereas, for the most unstable species, the CCSD(T)-F12b relative energies and structural parameters differ considerably from those computed using the B2PLYPD3 method.

**Table 1:** Relative Energies Including ZPV Energies (in kcal mol^–1^ and Also in Parentheses in eV), Equilibrium Rotational Constants (in MHz), and Dipole Moment Components (in D) for the Different [CH_3_, P, C, O] Isomers

	method	Δ*E*	*A*_e_	*B*_e_	*C*_e_	μ_a_	μ_b_	μ_c_	μ
CH_3_PCO	B2PLYPD3	0.00 (0.00)	14,640.5	3926.2	3158.0	1.43	–1.04	0.00	1.77
	CCSD(T)-F12b	0.00 (0.00)	14,663.2	3959.2	3180.5				
CH_3_OCP	B2PLYPD3	28.72 (1.25)	35,812.0	2920.7	2747.7	2.60	–1.17	0.0	2.85
	CCSD(T)-F12b	28.01 (1.21)	34,944.4	2955.5	2773.3				
CH_3_CPO	B2PLYPD3	46.50 (2.02)	81,498.0	2873.2	2824.8	3.67	–0.83	0.00	3.77
	CCSD(T)-F12b	41.20 (1.79)	77,924.2	2898.4	2844.9				
CH_3_COP	B2PLYPD3	93.20 (4.04)	37,750.9	2973.0	2804.3	1.88	–1.20	0.00	2.23
	CCSD(T)-F12b	88.88 (3.85)	45,719.6	2811.1	2693.2				
CH_3_OPC	B2PLYPD3	97.82 (4.24)	13,858.8	5193.9	3870.8	2.71	–0.45	0.00	2.75
	CCSD(T)-F12b	91.59 (3.97)	14,000.1	5249.3	3912.9				

Based on our computations, the five isomers show a bent heavy atom skeleton (see Figure 1). The most stable structure, CH_3_PCO, features the −PCO moiety linked to the –CH_3_ group through the P atom at an angle of ∠CPC = 95.8° [at the CCSD(T)-F12b level]. When the –CH_3_ group is connected to this moiety *via* the O atom instead of P, we found a less stable isomer, CH_3_OCP [∠COC = 114.1° at the CCSD(T)-F12b level], located at 28.01 kcal mol^–1^ [at the CCSD(T)-F12b level] above the global minimum. Following in energy, we found two structures with the –CH_3_ group connected through the C atom, the quasi-linear (zigzag CCPO structure) CH_3_CPO [∠CCP = 146.0° at the CCSD(T)-F12b level] and CH_3_COP [∠CCO = 122.8° at the CCSD(T)-F12b level], which lay at 41.20 and 88.88 kcal mol^–1^ [at the CCSD(T)-F12b/cc-pVTZ-F12 level], respectively, above CH_3_PCO. The less stable structure is CH_3_OPC, showing an energy difference with respect to CH_3_PCO of 91.59 kcal mol^–1^. In summary, regardless of the level of the theory used, we obtain the following stability order: CH_3_PCO > CH_3_OCP > CH_3_CPO > CH_3_COP > CH_3_OPC (where “>” means more stable than). This energetic trend is approximately in line with the results reported for the analogous CH_3_NCO isomers in a previous theoretical study by Dalbouha *et al.* in 2016 (CH_3_NCO > CH_3_OCN > CH_3_CNO > CH_3_ONC).^61^ However, Dalbouha *et al.* found that CH_3_CON is a transition state when CCSD(T)-F12 is considered and only behaves as an equilibrium structure when the MP2 methodology is used.

Moreover, we observe some dissimilarities between the structures of the high-energy isomers containing N and P. First, the heavy atom skeleton of CH_3_OPC is radically different than that of CH_3_ONC, showing a bent OPC moiety [∠OPC = 115.7° at the CCSD(T)-F12b level] compared to the linear ONC moiety of CH_3_ONC.^61^ Also, contrary to the zigzag structure of CH_3_CPO (see Figure 1), the skeleton of CH_3_CNO is completely linear (*C*_3*v*_ species).

The nature of the chemical bonding was characterized through the topological analysis of the electron charge density distribution, ρ(*r*), in the framework of QTAIM.^56^ The local properties of the electronic charge distribution for the [CH_3_, P, C, O] isomers are summarized in Tables S1–S5 of the Supporting Information. In addition, the contour maps of the Laplacian of the electron density including the molecular graph of the isomers are displayed in Figures S1–S5 of the Supporting Information. The two bonds concerning the phosphorus atom, P–C, P–O, have relatively small values of electron charge density and positive values of its Laplacian. Thus, they can be classified, in principle, as closed-shell interactions. However, the total energy density is negative (small in absolute value) and the value of the relationship between the local kinetic energy density and the local potential energy density is between 1 and 2. Thus, P–O and P–C bonds can be classified as intermediate interactions, mainly of covalent character, with a certain degree of ionic nature. Both the shared-shell character of the C–C, C–O, and C–H bonds and the intermediate characteristics of the P–O and P–C bonds can be visualized in the contour maps of the Laplacian (Figures S1–S5).

Regarding C–O bonds, we observe some dissimilarities in the local topological properties among the various isomers. Thus, in CH_3_PCO the electron charge density distribution is greater than in both CH_3_OCP and CH_3_COP, in line with the optimized C–O bond distances. Similarly, for the C–P bonds, ρ(*r*) is smaller in both the CH_3_PCO and CH_3_OPC isomers than in the CH_3_OCP and CH_3_CPO ones, reflecting again the differences in the C–P bond distances.

To analyze the stability of the [CH_3_, P, C, O] isomers, we have computed their adiabatic dissociation energies. For this purpose, we have considered, for each isomer, the process leading to a methyl radical and the corresponding [P, C, O] unit. In Table 2, we have collected the dissociation energies calculated at the B2PLYPD3/aug-cc-pVTZ and CCSD(T)-F12b/cc-pVTZ-F12 levels. As shown in Table 2, all adiabatic dissociation energies are positive and, thus, the [CH_3_, P, C, O] isomers will be stable against fragmentation. At first sight, slightly smaller values of the dissociation energies are obtained with the B2PLYPD3 methodology compared to those predicted at the CCSD(T)-F12b level. Nevertheless, we observe an overall good agreement between the dissociation energies computed at the two levels of the theory employed. The highest dissociation energy was obtained for the fragmentation of CH_3_CPO to give CH_3_ and CPO [102.13 kcal mol^–1^ (4.43 eV) at the CCSD(T)-F12b level]. Following in energy, we find the adiabatic dissociation associated with the generation of CH_3_ and PCO from the most stable isomer, CH_3_PCO [64.24 kcal mol^–1^ (2.79 eV) at the CCSD(T)-F12b level of theory]. These results suggest that both CH_3_CPO and CH_3_PCO isomers are the most stable ones against fragmentation into CH_3_ and either CPO or PCO. In contrast, the stability upon dissociation of CH_3_OCP and CH_3_COP to give CH_3_ and either PCO or COP is relatively low, with dissociation energies of 36.23 kcal mol^–1^ (1.57 eV) and 39.91 kcal mol^–1^ (1.73 eV) respectively.

**Table 2:** Dissociation Energies (*D*_0_), in kcal mol^–1^ (eV), Computed at the B2PLYPD3/aug-cc-pVTZ and the CCSD(T)-F12b/cc-pVTZ-F12 Levels of Theory^a^

reaction	B2PLYPD3 *D*_0_/kcal mol^–1^ (eV)	CCSD(T)-F12b *D*_0_/kcal mol^–1^ (eV)
CH_3_PCO → CH_3_ + PCO	61.58 (2.67)	64.24 (2.79)
CH_3_OCP → CH_3_ + PCO	32.86 (1.42)	36.23 (1.57)
CH_3_CPO → CH_3_ + CPO	101.42 (4.40)	102.13 (4.43)
CH_3_COP → CH_3_ + COP	36.23 (1.57)	39.91 (1.73)
CH_3_OPC → CH_3_ + CPO	50.10 (2.17)	51.74 (2.24)

a Zero-point vibrational energy corrections are included.

Also, as shown in Table 2, the stability of the [CH_3_, P, C, O] isomers does not correlate with their stability against dissociation. Instead, the stability of the distinct [P, C, O] fragments or units emerges as the driving factor in the energy stabilization of the parent species. In the two most stable structures, namely CH_3_PCO and CH_3_OCP, the CH_3_ group is linked to the PCO moiety (either through the P or the O atom) that is the most stable structure among the [P, C, O] isomers. Similarly, the greater stability against fragmentation of the CH_3_CPO isomer is a consequence of the lower stability of the CPO radical.

### 3.2 Analysis of the Methyl Internal Rotation

**Figure 2:**
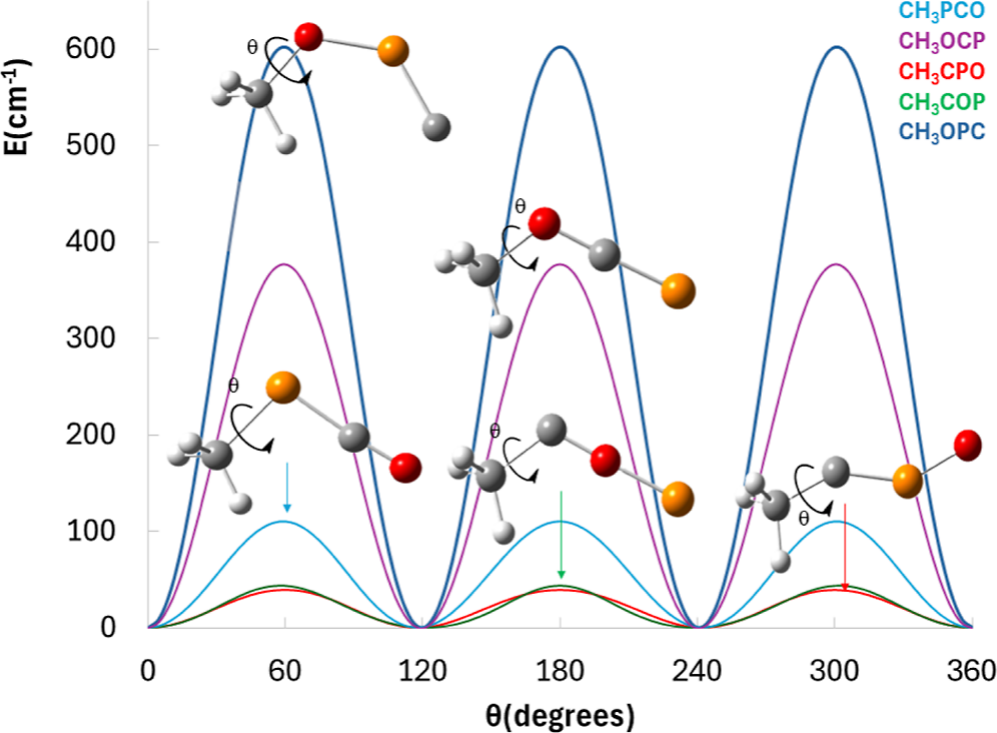
Relaxed potential energy surface (PES) scan computed at the B2PLYPD3/aug-cc-pVTZ level, choosing the ∠HCXY (where X,Y = O, P, or C) torsion as the driving coordinate.

As shown in Figure 1, all the studied isomers exhibit a –CH_3_ group, whose internal rotation, if feasible, will couple with the overall rotation of the molecule. This rotation phenomenon has relevant implications in the structure but also for the reactivity of the studied molecules.^62^ Particularly, depending on the magnitude of the *V*_3_ torsional barrier height, this motion may originate sizable *A*–*E* torsional splittings (fine structure) in the rotational spectra corresponding to the ground state of the molecule,^63^ whose analysis will be essential to achieve a proper experimental characterization. Hence, we have computed the *V*_3_ barrier height associated with the methyl internal rotation for each structure to evaluate the occurrence of possible *A*–*E* doublets. To do so, we have performed five relaxed energy scans, choosing the HCXY torsional angle (where X,Y = P, O, or C) as the driving coordinate to study the methyl internal rotation at the B2PLYPD3/aug-cc-pVTZ and CCSD(T)-F12b/cc-pVTZ-F12//B2PLYPD3/aug-cc-pVTZ levels of theory. The results are shown in Figure 2, while the calculated torsional barrier heights are collected in Table 3.

**Table 3:** Methyl Group Torsional Barriers for the [CH_3_, P, C, O] Isomers Computed at the B2PLYP/aug-cc-pVTZ and CCSD(T)-F12b/cc-pVTZ-F12//B2PLYPD3/aug-cc-pVTZ Levels of Theory

	B2PLYPD3 Δ(*E*)/cm^–1^	CCSD(T)-F12b//B2PLYPD3 Δ(*E*)/cm^–1^
CH_3_PCO	111	82
CH_3_OCP	377	405
CH_3_CPO	39	22
CH_3_COP	44	230
CH_3_OPC	602	524

We obtain values ranging from 22 to 524 cm^–1^ [CCSD(T)-F12b/cc-pVTZ-F12//B2PLYPD3/aug-cc-pVTZ level]. These values will be translated in a splitting of a few MHz for the intermediate barrier heights (*i.e.*, *V*_3_ = 405 cm^–1^ for CH_3_OCP), while the expected splitting for both CH_3_COP and CH_3_CPO will be much larger, further complicating the analysis of their rotational spectra. However, in the case of CH_3_OPC, this internal motion will be slightly more hindered by the crowded environment of the methyl group (see Figure 1).

Moreover, we can compare these results with the barrier heights previously computed for the N-analogs:^61^ methyl isocyanate *V*_3_(CH_3_NCO) = 16.2 cm^–1^; methyl cyanate *V*_3_(CH_3_OCN) = 364.8 cm^–1^; methyl fulminate *V*_3_(CH_3_ONC) = 821.7 cm^–1^. We found similarities between CH_3_OCP and CH_3_OCN. Meanwhile, the barrier height value obtained for CH_3_PCO is larger than the effective barrier found for CH_3_NCO, yet the barrier is still very low [*V*_3_(CH_3_PCO) = 82 cm^–1^], and the barrier found for CH_3_OPC is considerably smaller than the effective barrier of CH_3_ONC [*V*_3_(CH_3_OPC) = 524 cm^–1^]. This result is not striking and can be easily rationalized in terms of the different structures of both CH_3_OPC (see Figure 1) and CH_3_ONC.^61^ Regarding CH_3_PCO, the obtained value will slightly ease its spectral identification compared to the analysis of the N-analog, which can be essentially portrayed as a free rotor.^38,61,64^ Finally, for CH_3_COP and CH_3_CPO, we obtain extremely low torsional barrier heights, which will imply that both structures can also be considered as free rotors.

These results are also consistent with the values obtained for related CH_3_-bearing systems exhibiting different chemical environments for the methyl group: methylamine, *V*_3_(CH_3_NH_2_) = 708.6 cm^–1^,^65^ acetaldehyde, *V*_3_(CH_3_CHO) = 412.7 cm^–1^,^66^ methanol, *V*_3_(CH_3_OH) = 377.9 cm^–1^,^67^ acetone *V*_3_(CH_3_C(O)CH_3_) = 267 cm^–1^,^68^ dimethyl ether *V*_3_(CH_3_OCH_3_) = 950.6 cm^–1^,^69^ or the more complex *cis*–*trans n*-butanal *V*_3_(CH_3_(CH_2_)_2_CHO) = 1067 cm^–1^,^70^ and the glycine isomer *Z*-acetohydroxamic acid, *V*_3_ (CH_3_CONH_2_OH) = 255.4 cm^–1^.^71^

### 3.3 Spectroscopic Parameters for Laboratory Detection by Means of Rotational or Infrared Spectroscopy

In this section, we provide a complete set of the relevant rotational spectroscopic constants for the most stable isomer, CH_3_PCO. This isomer appears as the most promising species from an astrochemical point of view, and our aim is to ease eventual spectral searches. We present in Table 4 the equilibrium rotational constants (*A*, *B*, and *C*), calculated at the CCSD(T)-F12b/cc-pVTZ-F12 level of theory, along with the ground state spectroscopic constants (*A*_0_, *B*_0_, and *C*_0_), which incorporate the vibrational contribution or correction, derived from the vibration–rotation coupling parameters (computed from the full anharmonic CFF) and the degeneracy factors of the vibrational modes. We note that equilibrium rotational constants calculated at the CCSD(T)-F12b/cc-pVTZ-F12 level are found in excellent agreement with those obtained using the same “composite” scheme employed in Redondo *et al.*,^53^ which includes corrections to account for basis set truncation error, diffuse function, and core–valence correlation. The dipole moment components, computed at the CCSD/aug-cc-pVTZ level, are also shown in Table 4. These values, while not excessively large, are sizable enough to enable an eventual characterization through rotational spectroscopy. Additionally, we list in Table 4 the quartic centrifugal distortion constants considering the *S*-reduced form of an asymmetric Hamiltonian in the *I*^r^ representation, *H*_R_^(*A*)^.^72^

**Table 4:** Theoretically Predicted Rotational Parameters for the Most Stable Isomer CH_3_PCO, (*S*-Reduction, I^r^-Representation)

	CH_3_PCO
*A*^a^	14,663.23
*B*	3959.17
*C*	3180.47
*A*_0_^b^	14,604.75
*B*_0_	3950.27
*C*_0_	3165.63
*D*_J_ × 10^3^	2.56
*D*_JK_ × 10^3^	–5.83
*D*_K_ × 10^3^	158
*d*_1_ × 10^5^	–92.9
*d*_2_ × 10^5^	–8.25
|μ_a_|/|μ_b_|/|μ_c_|^c^	1.33/1.03/0.00

a *A*, *B*, and *C* are the equilibrium rotational constants computed at the CCSD(T)-F12b/cc-pVTZ-F12 level (in MHz).

b *A*_0_, *B*_0_, and *C*_0_ (in MHz) are the ground-state rotational constants computed considering CCSD(T)/cc-pVTZ anharmonic corrections.

c |μ_a_|, |μ_b_|, and |μ_c_| are the absolute values of the electric dipole moment components (in D) computed at the CCSD/aug-cc-pVTZ level.

At this point, before initiating the spectral assignment, we must address the fine structure of the molecule, whose resolution will be a puzzling task from both experimental and theoretical points of view. Nevertheless, we can neglect the internal rotation at first and treat CH_3_PCO as a rigid rotor (only considering the *A*-symmetry species). Hence, we will initially target transitions belonging to the *A*-symmetry substate, particularly *a*-type *R*-branch transitions ascribable to the *K*_a_ = 0, 1 ladders. These transitions are expected to be the dominant features in the rotational spectra and should also be relatively easy to identify owing to their characteristic *B* + *C* spacing. This analysis will be followed by the search for several *b*-type *R*-branch lines, which are also allowed due to dipole moment selection rules (μ_b_ = 1.03 D). Afterward, we will hunt for transitions belonging to the *E*-symmetry substate. To enable this search, we employed the XIAM program,^73^ developed to treat the effect of internal rotation, which uses a combination of the principal axis method (PAM) and the rho axis method and will allow us to predict the location of transitions arising from both *A* and *E*-symmetry substates. This approach has been used successfully to treat the internal rotation effect of molecules exhibiting a similar *V*_3_ barrier height, *e.g.*, *n*-propyl acetate [CH_3_C(O)O(CH_2_)_2_CH_3_].^74^

For the XIAM computation, we employed the calculated internal rotation barrier, *V*_3_ = 82 cm^–1^ (see Section 3.2), and the CCSD(T)-F12b/cc-pVTZ-F12 computed structure including also the quartic centrifugal distortion constants in our predictions. Additionally, we assumed a pure 3-fold potential without any 6-fold and higher contributions. Due to the small barrier height, lines belonging to both *A*- and *E*-symmetry states are predicted to be considerably separated in frequency, especially for the *b*-type transitions, a fact that will significantly hamper their conclusive identification. We anticipate that large frequency scans should be carried out to find the above lines, although we can expect the same sort of *a*-type *R*-branch progressions. We report in Table 5 a preliminary sample list of transitions that are anticipated to be the easiest and most promising targets during the initial inspection of the spectra in a typical 8–20 GHz frequency window, as a guide to ease the analysis. Also, it should be noted that the *K*_a_ and *K*_c_ pseudo quantum numbers are only useful for designating the energy levels of the *A*-symmetry substate, while only the *K*_a_ quantum number is of significance in the case of the *E*-symmetry substate transitions.

**Table 5:** Sample List of the Transitions Predicted for the *A*- and *E*-Symmetry States of CH_3_PCO

*J*	*K*_a_	*K*_c_	*J*′	*K*_a_^′^	*K*_c_^′^	*v*(*E*)/GHz	*v*(*A*)/GHz
1	0	1	0	0	0	6.948564	7.169625
2	0	2	1	0	1	13.920848	14.295511
2	1	1	1	1	0	14.581480	15.147510
2	1	2	1	1	1	14.087176	13.530608
1	1	0	1	0	1	14.100361	11.606273
1	1	1	0	0	0	15.461987	17.967427
3	0	3	2	1	2	12.215556	11.302475
4	0	4	3	1	3	19.365516	19.281854
3	1	2	3	0	3	15.990699	13.816185
4	1	3	4	0	4	17.809971	15.770318
5	1	4	5	1	5	13.338127	12.084922

We note that XIAM may experience some troubles in evaluating internal rotations with very low barrier heights because the torsional interactions between different *v*_t_ states are not considered explicitly, and additional programs might exhibit a better performance specially to achieve a proper experimental fit of the rotational transitions. Nevertheless, the information provided here shall be useful as a preliminary theoretical inspection of the rotational spectrum of CH_3_PCO. Hence, it will be in great demand for the laboratory spectroscopic community and, eventually, for the astrophysical community. Interestingly, this sort of *A*–*E* fine structure attributed to the methyl internal rotation motion can be generally used as a molecular “fingerprint” to search for a molecule in an astronomical molecular line survey,^38,75^ once it has been previously characterized and fully resolved in the laboratory.

Finally, we report in Table 6 the harmonic, ω, and anharmonic, ν, vibrational frequencies and IR intensities for the CH_3_PCO isomer, computed at the CCSD(T)/aug-cc-pVTZ level. This theoretical information will be of great relevance to model a reliable IR spectrum and search for IR spectral features of this molecule. As shown in Table 6, the IR spectrum of CH_3_PCO is dominated by an intense band related to the σ-PCO asymmetric stretching mode (*I*_anhar_ = 716.1 km/mol) located at ν = 1973 cm^–1^ or 5.06 μm, which will likely be the main target in an eventual spectral search based on IR spectroscopy. For completeness, we report in Table S6 of the Supporting Information the harmonic, ω, vibrational frequencies, and IR intensities for all the [CH_3_, P, C, O] isomers calculated at the B2PLYPD3/aug-cc-pVTZ level and in Table S7 the harmonic vibrational frequencies at the CCSD(T)-F12b/cc-pVTZ level.

**Table 6:** Harmonic, ω, and Anharmonic, ν, Vibrational Frequencies and IR Intensities, for CH_3_PCO (^1^A′) Isomer

mode	ω (cm^–1^)^a^	*I*_har_ (km/mol)^a^	ν (cm^–1^)^b^	*I*_anhar_ (km/mol)^b^
a″	60	0.0	47	0.0
a′	148	2.6	148	2.5
a″	452	0.0	450	0.0
a′	534	1.8	529	1.6
a′	655	1.8	640	1.6
a′	709	3.9	695	4.1
a″	887	2.7	876	2.6
a′	920	7.2	905	4.3
a′	1318	12.0	1395	7.4^c^
a″	1482	5.7	1442	4.8
a′	1488	4.3	1442	2.6
a′ (σ-PCO asymmetric stretching)	2003	716.1	1973	627.5
a′	3050	13.7	2937	12.1
a′	3136	3.6	2991	5.0
a″	3150	2.5	3002	4.5

a Harmonic vibrational frequencies and intensities calculated at the CCSD(T)/aug-cc-pVTZ level.

b Anharmonic contributions are computed at the CCSD(T)/cc-pVTZ level.

c Anharmonic contribution is computed at the CCSD(T)/aug-cc-pVDZ level.

## 4 Conclusions

In summary, we have performed a theoretical investigation on the [CH_3_, C, P, O] isomeric system using double-hybrid, B3LYPD3, and explicitly correlated coupled cluster, CCSD(T)-F12b methodologies. We have identified five distinct isomers, namely CH_3_PCO, CH_3_OCP, CH_3_CPO, CH_3_COP, and CH_3_OPC, all of them exhibiting a bent heavy atom skeleton, and analyzed their stability and dissociation processes. Additionally, we present a detailed topological analysis using QTAIM for the five isomers to characterize the nature of the chemical bonding. Among all structures, CH_3_PCO stands as the most stable species; consequently, it will be the main target of our theoretical spectroscopic characterization. We report an initial set of rotational spectroscopic parameters, which shall be used as primary data to guide eventual spectral assignments. Also, we have computed the hindering torsional barrier of the –CH_3_ methyl group for all structures. We find barrier heights ranging from very low (22 cm^–1^) to moderate values (524 cm^–1^), further providing preliminary information on the methyl internal rotation motion. Particularly, for CH_3_PCO we found a *V*_3_ = 82 cm^–1^, which is slightly larger than the extremely low barrier height derived experimentally for the N-analog species, CH_3_NCO, yet it is still very low. Afterward, we employed the XIAM program to provide a preliminary sample list of the most relevant rotational transitions from an experimental/laboratory point of view. Nevertheless, further theoretical and experimental effort will likely be needed to properly treat the internal rotation motion of this molecule and derive accurate transition frequencies. Finally, we provide complementary theoretical information on the IR spectra of all isomers, including CCSD(T)/aug-cc-pVTZ harmonic vibrational frequencies and anharmonic corrections estimated at the CCSD(T)/cc-pVTZ level for CH_3_PCO.

## Supporting Information Available

Local properties of the electronic charge distribution, contour maps of the Laplacian of the electron density including the molecular graph, harmonic vibrational frequencies, and IR intensities for all the [CH_3_, P, C, O] isomers (PDF)
