# Supplementary material for: Structure and Spectroscopic Insights for CH3PCO Isomers: A High-Level Quantum Chemical Study
Source: J Phys Chem A. 2024 May 9;128(20):4083–91. doi: 10.1021/acs.jpca.4c01370 (PMC11129311; doi:10.1021/acs.jpca.4c01370)

Supporting Information for

Structure and Spectroscopic Insights for CH_3PCO Isomers: a High-Level Quantum Chemical Study

Miguel Sanz-Novo[a], Pilar Redondo[b], Clara Isabel Sánchez[b], Antonio Largo[b], Carmen Barrientos[b] and José Ángel Sordo[c*]

[a]Centro de Astrobiología (CAB), INTA-CSIC, Carretera de Ajalvir km 4, Torrejón de Ardoz, 28850 Madrid, Spain

[b]Departamento de Química Física, Universidad de Valladolid, 47011 Valladolid, Spain

[c]Departamento de Química Física y Analítica, Laboratorio de Química Computacional, Facultad de Química, Universidad de Oviedo, Julián Clavería 8, 33006 Oviedo, Principado de Asturias, Spain

*Corresponding author

Content of this file:

Table S1: Local Topological Properties of the Electronic Charge Density Distribution for the CH_3PCO isomer

Table S2: Local Topological Properties of the Electronic Charge Density Distribution for the CH_3OCP isomer

Table S3: Local Topological Properties of the Electronic Charge Density Distribution for the CH_3CPO isomer

Table S4: Local Topological Properties of the Electronic Charge Density Distribution for the CH_3COP isomer

Table S5: Local Topological Properties of the Electronic Charge Density Distribution for the CH_3OPC isomer

Table S6: Harmonic, ω. Vibrational Frequencies (cm^{-1}) and IR Intensities (km/mol), for the [CH_3, C, P, O] isomers calculated at the B2PLYPD3/aug-cc-pVTZ level.

Table S7: Harmonic, ω. Vibrational Frequencies (cm^{-1}) for the [CH_3, C, P, O] isomers calculated at the CCSD(T)-F12**b**// cc-pVTZ-F12 level.

Figure S1: Contour maps of the Laplacian distribution of the electron density for the CH_3PCO isomer.

Figure S2: Contour maps of the Laplacian distribution of the electron density for the CH_3OCP isomer.

Figure S3: Contour maps of the Laplacian distribution of the electron density for the CH_3CPO isomer.

Figure S4: Contour maps of the Laplacian distribution of the electron density for the CH_3COP isomer.

Figure S5: Contour maps of the Laplacian distribution of the electron density for the CH_3OPC isomer.

Supplementary Tables:

Table S1:

Local Topological Properties (in au.) of the Electronic Charge Density Distribution Calculated at the B2PLYPD3 level, at the Position of the Bond Critical Points for the CH_3PCO isomer.[a]

Bond	$\boldsymbol{\rho(r)}$	$\boldsymbol{\nabla^2\rho(r)}$	$\boldsymbol{|V(r)|/G(r)|}$	$\boldsymbol{H(r)}$
C3 - O4	0.459727	0.284385	1.92202992	-0.840741
P1 - C3	0.160286	0.521456	1.49813481	-0.129395
C2 - H6	0.28897	-1.133298	8.03444016	-0.330275
C2 - H7	0.28897	-1.133298	8.03444016	-0.330275
C2 - H5	0.286342	-1.111343	7.78871156	-0.325832
P1 - C2	0.144532	-0.284294	3.31558196	-0.125097

Notes. $\rho(r)$: Electronic charge density; $\nabla^2\rho(r)$: Laplacian of electronic charge density; $|V(r)|/G(r)$: Relationship between the local kinetic energy density $G(r)$ and the local potential energy density $V(r)$; $H(r)$: Total energy density.

Table S2:

Local Topological Properties (in au.) of the Electronic Charge Density Distribution Calculated at the B2PLYPD3 level, at the Position of the Bond Critical Points for the CH_3OCP isomer.[a]

Bond	$\boldsymbol{\rho(r)}$	$\boldsymbol{\nabla^2\rho(r)}$	$\boldsymbol{|V(r)|/G(r)|}$	$\boldsymbol{H(r)}$
O1 - C3	0.339055	-0.302789	2.1581363	-0.554379
O1 - C2	0.230113	-0.344704	2.37637029	-0.315142
C3 - P4	0.192057	0.883204	1.41902577	-0.159252
C2 - H5	0.300248	-1.23421	10.0112424	-0.347068
C2 - H6	0.297339	-1.207082	9.71240544	-0.340899
C2 - H7	0.297339	-1.207082	9.71240544	-0.340899

Notes. $\rho(r)$: Electronic charge density; $\nabla^2\rho(r)$: Laplacian of electronic charge density; $|V(r)|/G(r)$: Relationship between the local kinetic energy density $G(r)$ and the local potential energy density $V(r)$; $H(r)$: Total energy density.

Table S3:

Local Topological Properties (in au.) of the Electronic Charge Density Distribution Calculated at the B2PLYPD3 level, at the Position of the Bond Critical Points for the CH_3CPO isomer.[a]

Bond	$\rho(r)$	$\nabla^2\rho(r)$	$\lvert V(r)\rvert/G(r)\lvert$	$H(r)$
O1 - P2	0.219992	1.251602	1.36905782	-0.183025
P2 - C3	0.196103	0.574309	1.55868494	-0.181762
C4 - H6	0.287472	-1.128031	8.28329843	-0.326889
C4 - H7	0.287472	-1.128031	8.28329843	-0.326889
C4 - H5	0.281806	-1.087709	7.93750819	-0.317725
C3 - C4	0.26657	-0.762536	4.34590158	-0.271897

Notes. $\rho(r)$: Electronic charge density; $\nabla^2\rho(r)$: Laplacian of electronic charge density; $|V(r)|/G(r)$: Relationship between the local kinetic energy density $G(r)$ and the local potential energy density $V(r)$; $H(r)$: Total energy density.

Table S4:

Local Topological Properties (in au.) of the Electronic Charge Density Distribution Calculated at the B2PLYPD3 level, at the Position of the Bond Critical Points for the CH_3COP isomer.[a]

Bond	$\rho(r)$	$\nabla^2\rho(r)$	$\lvert V(r)\rvert/G(r)\lvert$	$H(r)$
O2 - C3	0.344289	0.912522	1.70233909	-0.538279
P1 - O2	0.140462	0.925817	1.25312204	-0.07844
C4 - H5	0.293662	-1.176431	8.73368134	-0.337785
C4 - H6	0.280471	-1.07588	7.92677714	-0.314352
C4 - H7	0.280471	-1.07588	7.92677714	-0.314352
C3 - C4	0.25777	-0.691369	4.36927021	-0.245795

Notes. $\rho(r)$: Electronic charge density; $\nabla^2\rho(r)$: Laplacian of electronic charge density; $|V(r)|/G(r)$: Relationship between the local kinetic energy density $G(r)$ and the local potential energy density $V(r)$; $H(r)$: Total energy density.

Table S5:

Local Topological Properties (in au.) of the Electronic Charge Density Distribution Calculated at the B2PLYPD3 level, at the Position of the Bond Critical Points for the CH_3OPC isomer.[a]

Bond	$\boldsymbol{\rho(r)}$	$\boldsymbol{\nabla^2\rho(r)}$	$\boldsymbol{\lvert V(r)\rvert/G(r)\rvert}$	$\boldsymbol{H(r)}$
O3 - C4	0.237377	-0.391029	2.42489373	-0.327831
C1 - P2	0.196198	0.242111	1.76611579	-0.198271
P2 - O3	0.167173	0.639059	1.45383937	-0.132758
C4 - H5	0.298927	-1.222306	9.8258509	-0.344623
C4 - H6	0.29512	-1.188359	9.42078182	-0.337125
C4 - H7	0.29512	-1.188359	9.42078182	-0.337125

Notes. $\rho(r)$: Electronic charge density; $\nabla^2\rho(r)$: Laplacian of electronic charge density; $|V(r)|/G(r)$: Relationship between the local kinetic energy density $G(r)$ and the local potential energy density $V(r)$; $H(r)$: Total energy density.

Table S6:

Harmonic, ω. Vibrational Frequencies (cm^{-1}) and IR Intensities (km/mol), for the [CH_3, C, P, O] isomers calculated at the B2PLYPD3/aug-cc-pVTZ level.

mode	CH_3PCO		CH_3OCP		CH_3CPO		CH_3COP		CH_3OPC	
	ω	I_{har}	ω	I_{har}	ω	I_{har}	ω	I_{har}	ω	I_{har}
a’	149	2.6	183	6.3	189	5.9	158	15.5	99	35.4
a’	541	1.7	546	19.1	344	13.4	466	104.1	315	7.8
a’	649	1.4	756	13.7	696	8.1	664	3.9	739	62.3
a’	719	4.1	1003	147.8	998	2.1	951	208.6	945	38.7
a’	927	7.5	1209	9.1	1154	115.2	1073	45.7	1052	187.3
a’	1322	12.4	1458	71.1	1404	0.4	1360	90.8	1200	1.8
a’	1491	4.7	1509	9.3	1458	11.6	1461	0.0	1479	6.9
a’	2008	670.3	1691	488.7	1491	9.9	1525	471.0	1509	5.5
a’	3070	13.5	3070	39.3	3038	3.3	3007	16.9	3048	22.8
a’	3154	3.3	3194	7.9	3098	11.6	3182	5.3	3169	11.1
a″	83	0.0	139	0.7	64	8.0	15	0.0	155	4.4
a”	459	0.0	427	3.9	193	0.7	350	0.1	198	2.2
a”	891	3.0	1170	1.6	1042	0.0	896	3.8	1174	1.2
a″	1485	6.2	1503	12.0	1468	6.4	1485	13.7	1506	14.0
a”	3166	2.1	3157	14.1	3125	3.8	3070	8.4	3122	14.1

Table S7:

Harmonic, ω, Vibrational Frequencies (cm^{-1}) for the [CH_3, C, P, O] isomers calculated at the CCSD(F)-F12**b**/cc-pVTZ-F12 level.

mode	CH_3PCO	CH_3OCP	CH_3CPO	CH_3COP	CH_3OPC
a’	149	181	186	127	95
a’	538	545	346	401	323
a’	665	763	692	640	762
a’	721	1026	993	976	957
a’	919	1211	1185	1062	1079
a’	1319	1456	1397	1383	1201
a’	1482	1505	1446	1464	1475
a’	2015	1686	1487	1625	1503
a’	3052	3054	3033	3002	3032
a’	3140	3181	3103	3158	3159
a”	63	144	44	77	148
a”	456	422	187	383	196
a”	885	1174	1036	965	1177
a”	1478	1497	1466	1480	1501
a”	3154	3142	3130	3068	3110

Supplementary Figures:

Figure S1:

Contour maps of the Laplacian distribution of the electron density for the CH_3PCO isomer. Red dashed lines indicate regions of electronic charge concentration ($\nabla^2\rho(r)$< 0), and blue continuous lines denote regions of electronic charge depletion ($\nabla^2\rho(r)$> 0).

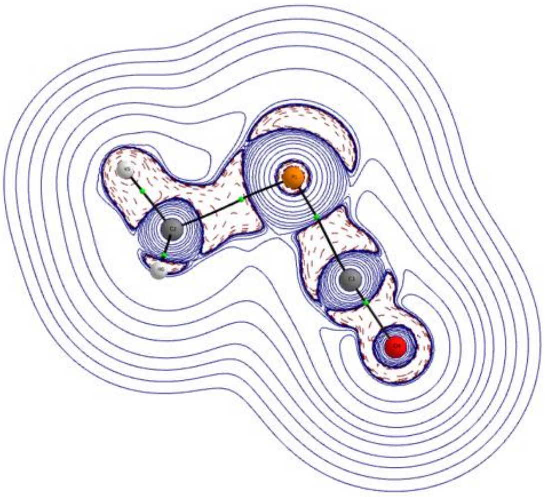

Figure S2:

Contour maps of the Laplacian distribution of the electron density for the CH_3OCP isomer. Red dashed lines indicate regions of electronic charge concentration ($\nabla^2\rho(r)$< 0), and blue continuous lines denote regions of electronic charge depletion ($\nabla^2\rho(r)$> 0).

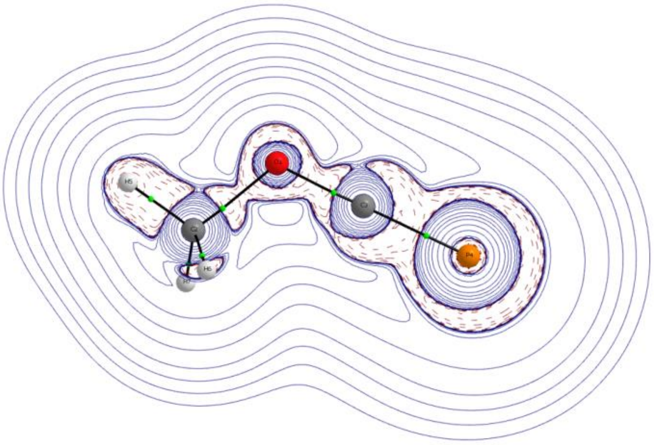

Figure S3:

Contour maps of the Laplacian distribution of the electron density for the CH_3CPO isomer. Red dashed lines indicate regions of electronic charge concentration ($\nabla^2\rho(r)$< 0), and blue continuous lines denote regions of electronic charge depletion ($\nabla^2\rho(r)$> 0).

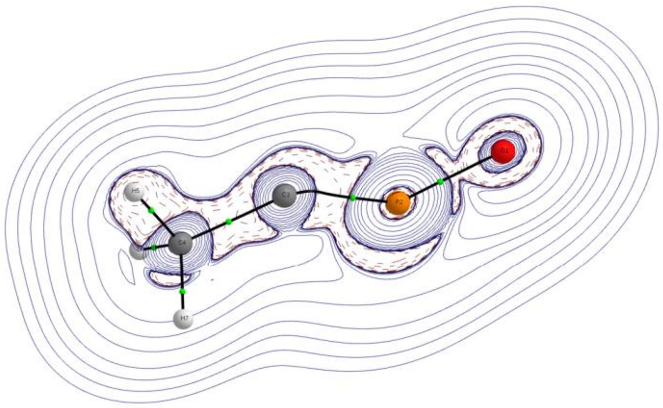

Figure S4:

Contour maps of the Laplacian distribution of the electron density for the CH_3COP isomer. Red dashed lines indicate regions of electronic charge concentration ($\nabla^2\rho(r)$< 0), and blue continuous lines denote regions of electronic charge depletion ($\nabla^2\rho(r)$> 0).

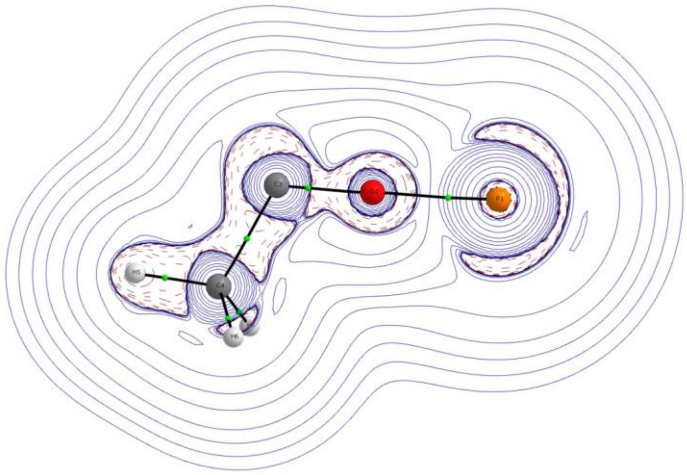

Figure S5:

Contour maps of the Laplacian distribution of the electron density for the CH_3OPC isomer. Red dashed lines indicate regions of electronic charge concentration ($\nabla^2\rho(r)<0$), and blue continuous lines denote regions of electronic charge depletion ($\nabla^2\rho(r)>0$).

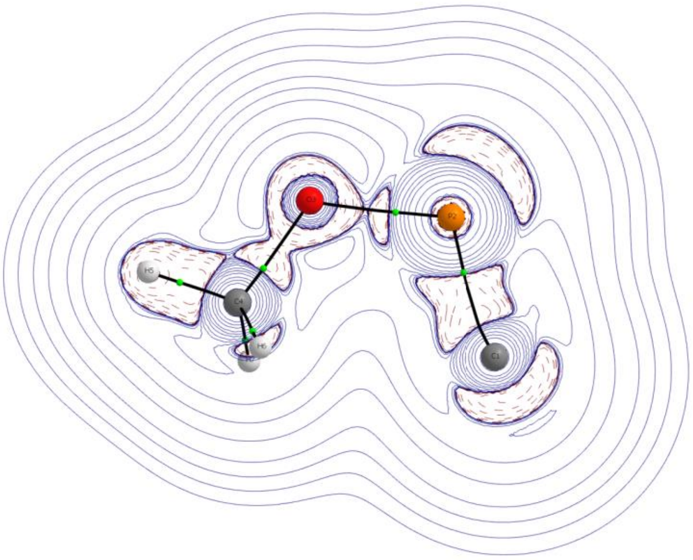

Supplement: Supplementary file 1 — jp4c01370_si_001.pdf [file jp4c01370_si_001.pdf]
